# Promoting Spatial Charge Transfer of ZrO_2_ Nanoparticles: Embedded on Layered MoS_2_/g-C_3_N_4_ Nanocomposites for Visible-Light-Induced Photocatalytic Removal of Tetracycline

**DOI:** 10.1021/acsomega.1c06089

**Published:** 2022-02-02

**Authors:** Elayaperumal Vijayakumar, Muniyandi Govinda Raj, Moorthy Gnanasekar Narendran, Rajaraman Preetha, Ramasamy Mohankumar, Bernaurdshaw Neppolian, Aruljothy John Bosco

**Affiliations:** † Department of Chemistry, SRM Institute of Science and Technology, Kattankulathur 603203, Tamil Nadu, India; ‡ Interdisciplinary Institute of Indian System of Medicine, SRM Institute of Science and Technology, Kattankulathur 603203, Tamil Nadu, India; § Energy and Environmental Remediation Laboratory, SRM Research Institute, SRM Institute of Science and Technology, Kattankulathur 603203, Tamil Nadu, India

## Abstract

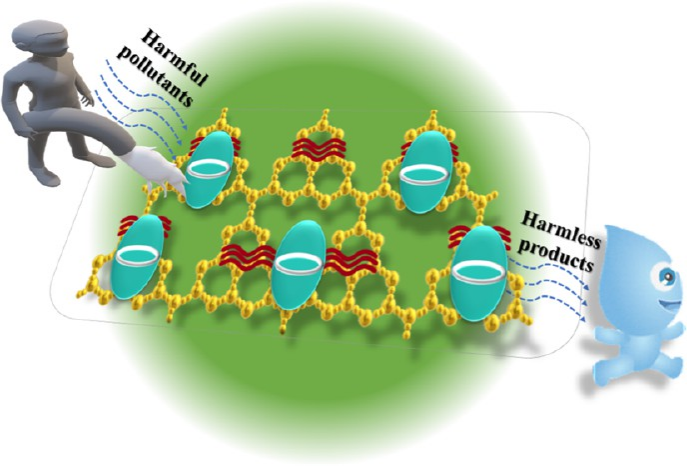

Photocatalytic degradation is a sustainable technique for reducing the environmental hazards created by the overuse of antibiotics in the food and pharmaceutical industries. Herein, a layer of MoS_2_/g-C_3_N_4_ nanocomposite is introduced to zirconium oxide (ZrO_2_) nanoparticles to form a “particle-embedded-layered” structure. Thus, a narrow band gap (2.8 eV) starts developing, deliberated as a core photodegradation component. Under optimization, a high photocatalytic activity of 20 mg/L TC at pH 3 with ZrO_2_@MoS_2_/g-C_3_N_4_ nanocomposite was achieved with 94.8% photocatalytic degradation in 90 min. A photocatalytic degradation rate constant of 0.0230 min^–1^ is determined, which is 2.3 times greater than the rate constant for bare ZrO_2_ NPs. The superior photocatalytic activity of ZrO_2_@MoS_2_/g-C_3_N_4_ is due to the dual charge-transfer channel between the MoS_2_/g-C_3_N_4_ and ZrO_2_ nanoparticles, which promotes the formation of photogenerated e^–^/h^+^ pairs. Charge recombination produces many free electron–hole pairs, which aid photocatalyst reactions by producing superoxide and hydroxyl radicals via electron–hole pair generation. The possible mechanistic routes for TC were investigated in-depth, as pointed out by the liquid chromatography–mass spectrometry (LC–MS) investigation. Overall, this work shows that photocatalysis is a feasible sorbent approach for environmental antibiotic wastewater treatment.

## 1 Introduction

In the past decade, antibiotics- and antiphlogistics-related pharmaceutical wastewater pollution has posed a hazard to human health and the environment. Tetracycline (TC) hydrochloride, a type of TC in general antibiotics, is mainly used in medicine, agriculture, and other fields, and it remains in the soil and groundwater. Improvements in TC can lead to the proliferation of drug-resistant microbes if they are exploited.^1−3^ Currently, many techniques have been developed for the removal of antibiotics from water environments, the most notable of which are photo-Fenton treatment and biological treatments such as ozonation and membrane filtration. Other techniques include electrochemical oxidation, semiconductor photocatalysis, and adsorption.^4−6^ Parallel to these classic technologies, semiconductor photocatalysis has gained significant attention in the field of antibiotic degradation due to its high efficiency and long-term sustainability, which is achieved through the use of solar light and ecologically favorable circumstances. Besides that, it can efficiently digest antibiotics and convert them into readily biodegradable composites with fewer harmful organic or inorganic compounds, diminishing or eliminating their antimicrobial effectiveness. It may be able to alleviate some of the issues associated with some orthodox techniques of antibiotic degradation, such as the problematic biodegradability of antibiotics and the possibility of secondary contamination caused by the intermediates.^7−10^

The most often utilized and investigated materials in heterogeneous photocatalysis are transition-metal oxides and wide-band semiconductors, such as TiO_2_, SnO_2_, CeO_2_, and ZrO_2_. In particular, the remarkable physicochemical stability of ZrO_2_ and the unique electronic energy band structure of this photocatalyst have piqued the curiosity of a large number of researchers. Because of their large band gap (>5 eV), ZrO_2_ photocatalysts can only absorb ultraviolet light, which represents a small percentage of the solar spectrum (less than 5%). As a result, the photocatalytic activity of ZrO_2_ is limited in its functional application as a photocatalyst. Apart from that, the poor separation rate between the charged particles generated by photons in ZrO_2_ restricts the photocatalytic degradation activity of the nanomaterial.^11^ Among the several approaches for overturning the enhancing light-harvesting and recombination of photogenerated carriers, coupling ZrO_2_ with another narrow-band-gap semiconductor, for example, TiO_2_, TiO_2_–ZrO_2_, g-C_3_N_4_, MoS_2_, etc.^12−14^

Two-dimensional (2D) layered semiconductors hold several remarkable characteristics, including rapid charge-carrier detachment, electronic conduction, and a vast surface area. It has been discovered that g-C_3_N_4_, a nonmetallic polymeric nanomaterial with optimal band-edge position (1.32 V, pH = 7) and a low band gap (2.8 eV), features moderate van der Waals forces between layers and low hydrogen-bonding connectivity bordered by polymeric melon units but strong covalent C–N bonds inside the melon units.^15^ g-C_3_N_4_ nanosheets can also help create nanocomposites with rich coupling heterointerfaces and surface-reactive positions. Studies on creating heterojunctions, including g-C_3_N_4_/ZrO_2_,^16^ TiOF_2_/g-C_3_N_4_,^17^ MoS_2_/g-C_3_N_4_/Bi_24_O_31_Cl_10_,^18^ and CsPbI_3_/g-C_3_N_4_,^19^ with the goal of overcoming the inherent disadvantages of g-C_3_N_4_, were conducted in-depth. This scheme reveals superior visible-light photoinduced activity in the direction of degradation compared to the solitary components.

Individual sandwiched S–Mo–S layers in molybdenum disulfide (MoS_2_) are intrinsic n-type photocatalysts with a narrow band gap (1.29–1.94 eV) and an anisotropic lamellar structure with weak van der Waals interactions between them. Molybdenum disulfide (MoS_2_) is a high-productivity cocatalyst for photocatalytic degradation due to the survival of unsaturated Mo and S atoms at the exposed edges, which are capable of encoring photocatalytic degradation. It also enhances visible-light absorption and minimizes reflection, allowing more free charge-transfer carriers for photocatalytic degradation.^20,21^ The layered structures of MoS_2_ and g-C_3_N_4_ help reduce lattice disparity and help form an electronic field at the interface of a 2D–2D nanocomposite, which aids in promoting charge separation and surface reactions. Thus, the nanocomposite charge separation and photoactivity degradation of the intended ZrO_2_@MoS_2_/g-C_3_N_4_ nanocomposite should be significantly enhanced.

In this work, as mentioned above, a feasible ultrasonic chemical technique was used for the fabrication of ZrO_2_@MoS_2_/g-C_3_N_4_ nanocomposites. Here, the ZrO_2_ nanoparticles (NPs) were embedded on the surface of 2D layered MoS_2_/g-C_3_N_4_ nanosheets (NSs). The band alignment of the composite was improved by employing a continuous multistep charge-carrier (e^–^/h^+^) transfer path rather than the standard one-step process. This study investigates the photocatalytic degradation of TC over ZrO_2_@MoS_2_/g-C_3_N_4_ using visible light. A minimum energy gap between the highest occupied molecular orbital (HOMO) and the lowest unoccupied molecular orbital (LUMO) exists due to the delocalized π link between the TC molecule and its connection to the −OH group, leading to the high availability for visible-light absorption. The TC’s π orbital may also create an electronic interaction with the 3d orbital of Zr^4+^, leading to a surface complex between TC and ZrO_2_. It is expected that visible-light irradiation will cause photoexcitation of the surface complex. The primary goals of this work are to confirm the presence of visible-light photodegradation of TC on ZrO_2_@MoS_2_/g-C_3_N_4_ and estimate the mechanism of photodegradation. Dependence on the ZrO_2_@MoS_2_/g-C_3_N_4_ nanocomposite, strong interface effects, relatively short charge-diffusion distance, and numerous close contact interfaces can be obtained simultaneously. The possible mechanistic routes for TC were investigated in-depth, as indicated by LC–MS analysis on the ZrO_2_@MoS_2_/g-C_3_N_4_ nanocomposites.

## 2 Results and Discussion

**Figure 1:**
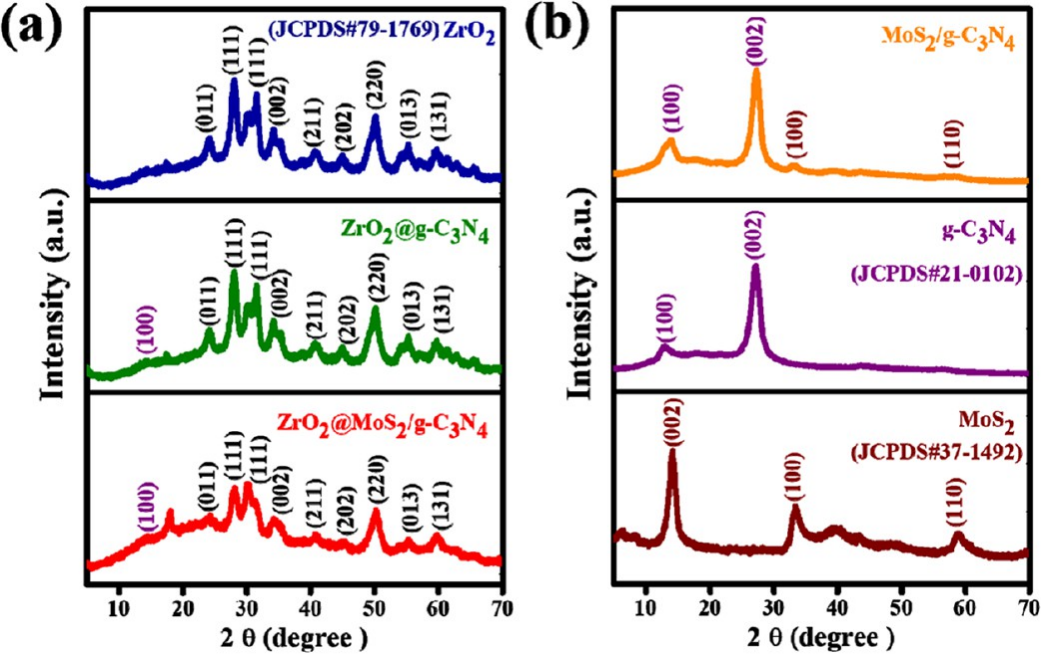
XRD patterns of (a) ZrO_2_, ZrO_2_@g-C_3_N_4_, ZrO_2_@MoS_2_/g-C_3_N_4_ and (b) MoS_2_, g-C_3_N_4_, MoS_2_/g-C_3_N_4_ nanocomposites.

XRD patterns of the as-synthesized products were obtained to confirm the crystal structure and the phase purity. As shown in Figure 1a,b, bare ZrO_2_ can be coded for tetragonal (JCPDS card 79-1769); its fundamental diffraction patterns at 24.2, 28.2, 31.5, 34.1, 35.3, 40.7, 50.1, and 55.3° can be assigned to (011), (111), (111), (002), (211), (202), (013), and (131) planes, respectively. The firm and spire diffraction peaks of ZrO_2_ confirm the high purity and crystallinity of the sample.^22,23^ The primary peaks of bare g-C_3_N_4_ are located at 13.1 and 27.4°, respectively, and correspond to the crystal planes (100) and (002).^24^ It has been observed that there are no distinctive peaks of g-C_3_N_4_ and MoS_2_ in the ZrO_2_@MoS_2_/g-C_3_N_4_ nanocomposite (Figure 5a,b) even though these peaks can be seen in the transmission electron microscopy (TEM) pictures. According to the results obtained for blank MoS_2_, the sequences of the diffraction pattern situated at 14.2, 33.2, and 58.9° are ascribable to the diffractions of (002), (100), and (110), corresponding to the usual hexagonal MoS_2_ structure (JCPDS card 37-1492). Remarkably, the addition of g-C_3_N_4_ and MoS_2_ had no adverse influence on the structure and purity of ZrO_2_ nanoparticles. However, the intensity of deflection points of ZrO_2_@MoS_2_/g-C_3_N_4_ is weaker and broader than that of pure ZrO_2_ due to the low crystallinity and the tiny crystallite size of the ZrO_2_ nanoparticles.

**Figure 2:**
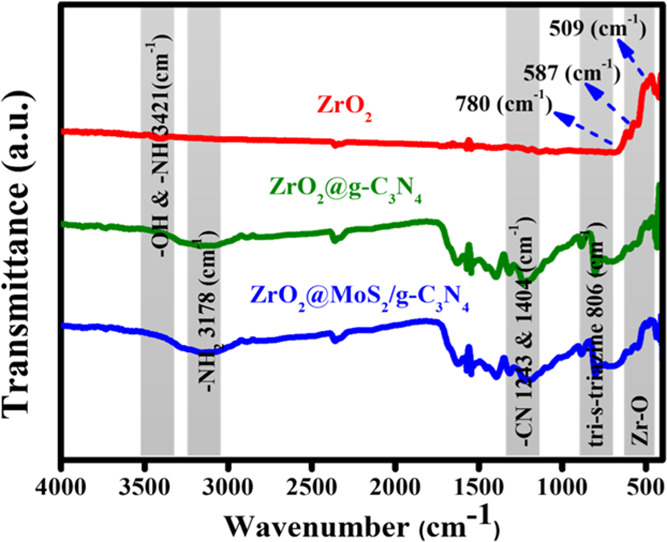
FT-IR spectra of ZrO_2_, ZrO_2_@g-C_3_N_4_, and ZrO_2_@MoS_2_/g-C_3_N_4_ nanocomposites.

FT-IR analysis was performed to investigate the surface chemical structure of as-prepared materials. As shown in Figure 2, all of the as-prepared ZrO_2_, ZrO_2_@g-C_3_N_4_, and ZrO_2_@MoS_2_/g-C_3_N_4_ nanocomposites materials retain not only the surface chemical structure of the ZrO_2_ nanoparticles but also the structure of the g-C_3_N_4_ semiconductor. These −NH_2_ stretching vibrations are responsible for the wide absorption bands in the 3178 cm^–1^ range. The peaks show the stretching vibration of C–N at 1243 and 1404 cm^–1^. The peak at 806 cm^–1^ corresponds to tri-s-triazine stretching vibrations. The bands at 507 and 587 cm^–1^ as well as 780 cm^–1^ correspond to the stretching vibration of the Zr–O bond.^25^

**Figure 3:**
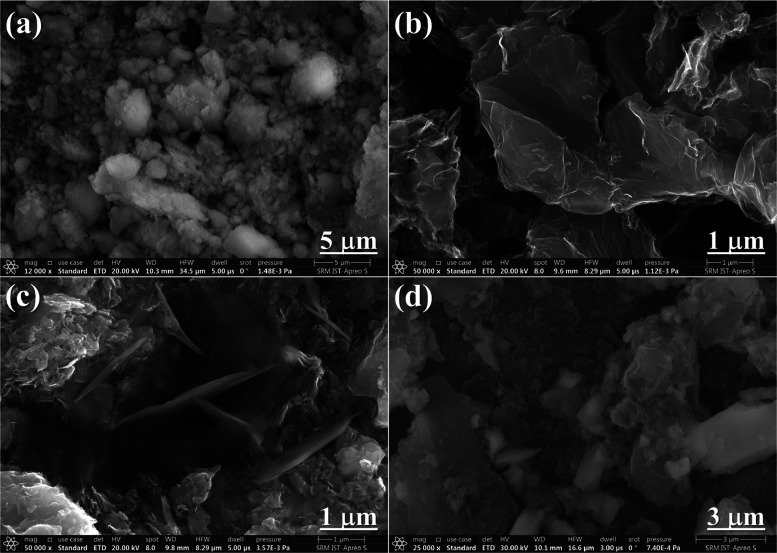
HR-SEM images of (a) ZrO_2_, (b) g-C_3_N_4_, (c) MoS_2_, and (d) ZrO_2_@MoS_2_/g-C_3_N_4_ nanocomposites.

**Figure 4:**
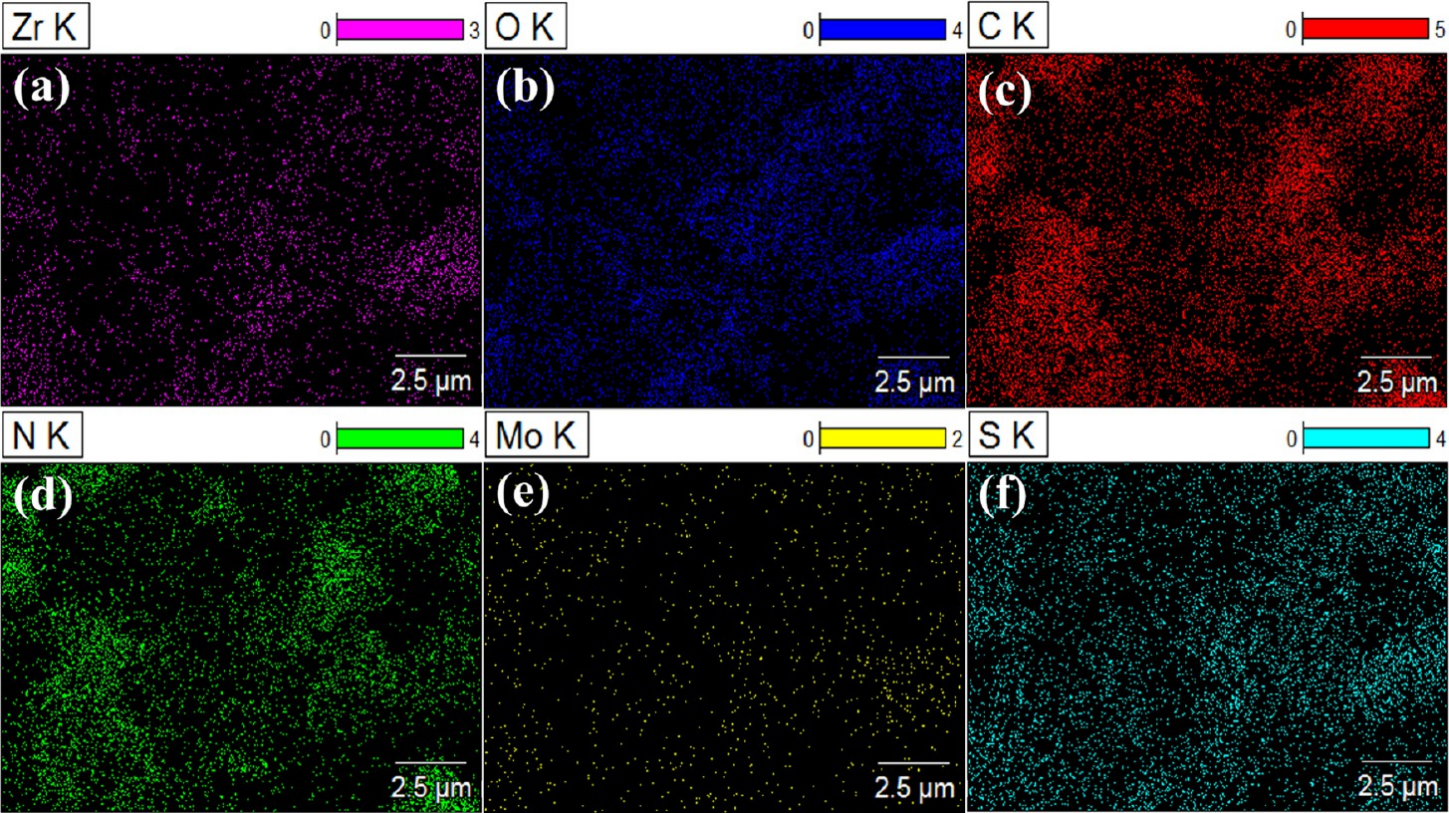
(a–f) Corresponding EDS mapping images of the ZrO_2_@MoS_2_/g-C_3_N_4_ nanocomposite.

Using HR-SEM, the surface morphologies and structural characteristics of ZrO_2_, g-C_3_N_4_, MoS_2_, and ZrO_2_@MoS_2_/g-C_3_N_4_ nanocomposite were studied. Figure 3a depicts the irregular spherical nanoparticles of ZrO_2_ with an average diameter of 15 nm, resulting in a large surface area of 43.2 m^2^ g^–1^. Due to its unique spherical structure, g-C_3_N_4_ has a small surface area of 13.5 m^2^ g^–1^ compared to pure ZrO_2_.^26^ A shape-free architecture is illustrated in Figure 3b, which has a smooth surface and is in the shape of a platelike structure composed of g-C_3_N_4_. It also reveals the strong interaction between the two ZrO_2_ and g-C_3_N_4_ semiconductors. Figure 3c clearly illustrates that pure MoS_2_ displays a hierarchical-like sheet with adjacent sizes of a limited micrometer and was piled sequentially.^27^ As shown in Figure 3d, the ZrO_2_@MoS_2_/g-C_3_N_4_ structure is not smooth and has grooves because this pattern is formed when ZrO_2_ nanoparticles are embedded, and more active sites and higher light absorption capacity might be obtained with a product including dispersed nanoparticles, which would be helpful to the formation of reactive free radicals. Figure 4a–f shows evenly distributed Zr, O, C, N, Mo, and S in ZrO_2_@MoS_2_/g-C_3_N_4_ with a “particle-embedded-layered” structure, demonstrating that the nanocomposite of ZrO_2_@MoS_2_/g-C_3_N_4_ was successfully constructed. The results are in line with the HR-SEM analysis.^28^

**Figure 5:**
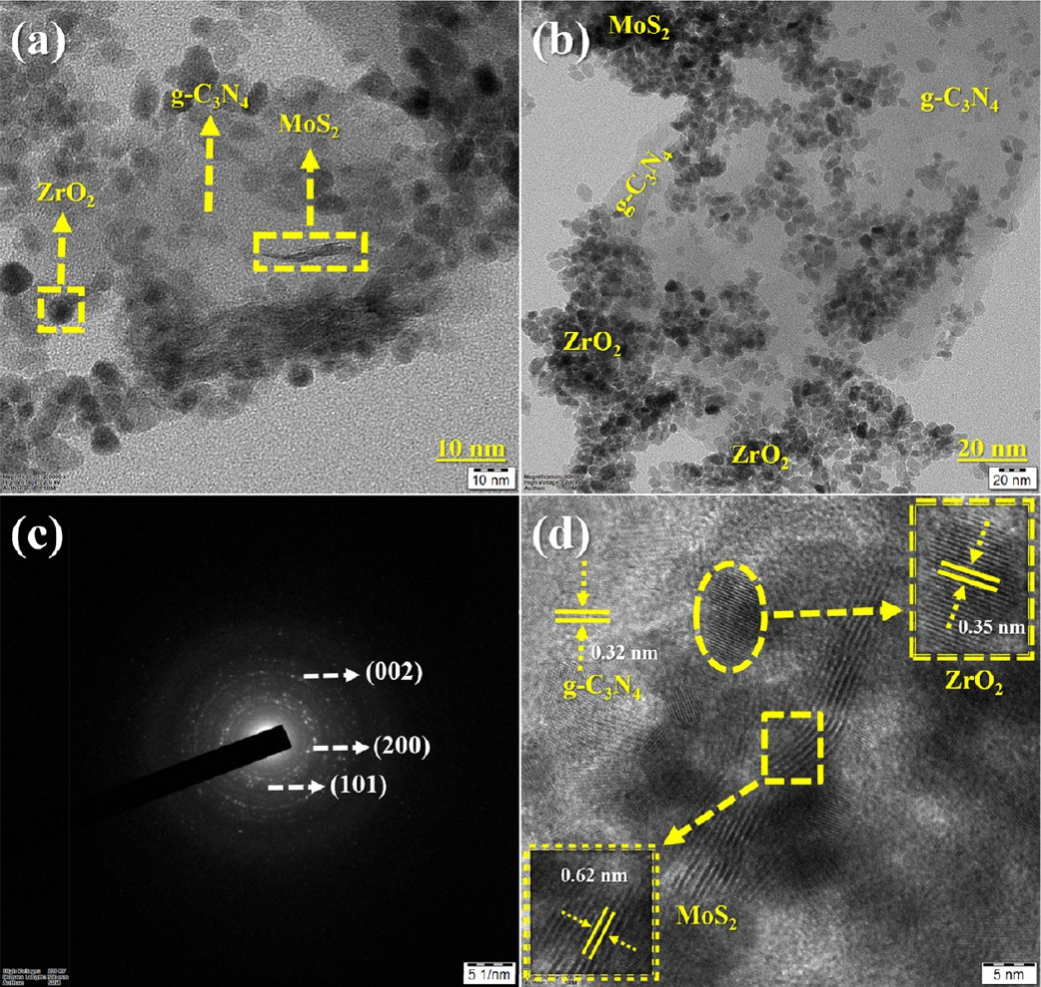
HR-TEM images of (a, b) different magnifications and (c, d) SAED patterns and lattice fringes of the ZrO_2_@MoS_2_/g-C_3_N_4_ nanocomposite.

The impact of inserting a 2D cocatalyst layered on recognition of nanocomposite creation using ZrO_2_ nanoparticles is established by HR-TEM scrutiny in Figure 5. The MoS_2_/g-C_3_N_4_ sheet in Figure 5a,b is embedded with ZrO_2_ nanoparticles with a regular diameter of 15 nm. The formation of spherical-shaped ZrO_2_ nanoparticles was confirmed by SAED analysis (Figure 5c). The XRD data show that the ZrO_2_@MoS_2_/g-C_3_N_4_ nanocomposite exhibits good crystallinity, and HR-TEM confirms the tetragonal structure of ZrO_2_ nanoparticles (101) (JCPDS no. 79-1769), which can be seen in Figure 5d. To define a single-phase growth, the lattice fringes show interplanar spacings (“*d*” values) of 0.35, 0.32, and 0.62 nm, which correspond to tetragonal ZrO_2_ (101), g-C_3_N_4_, and MoS_2_ (002) planes, respectively;^29−32^ therefore, the evidence presented above suggests that the ZrO_2_@MoS_2_/g-C_3_N_4_ nanocomposite has formed.

**Figure 6:**
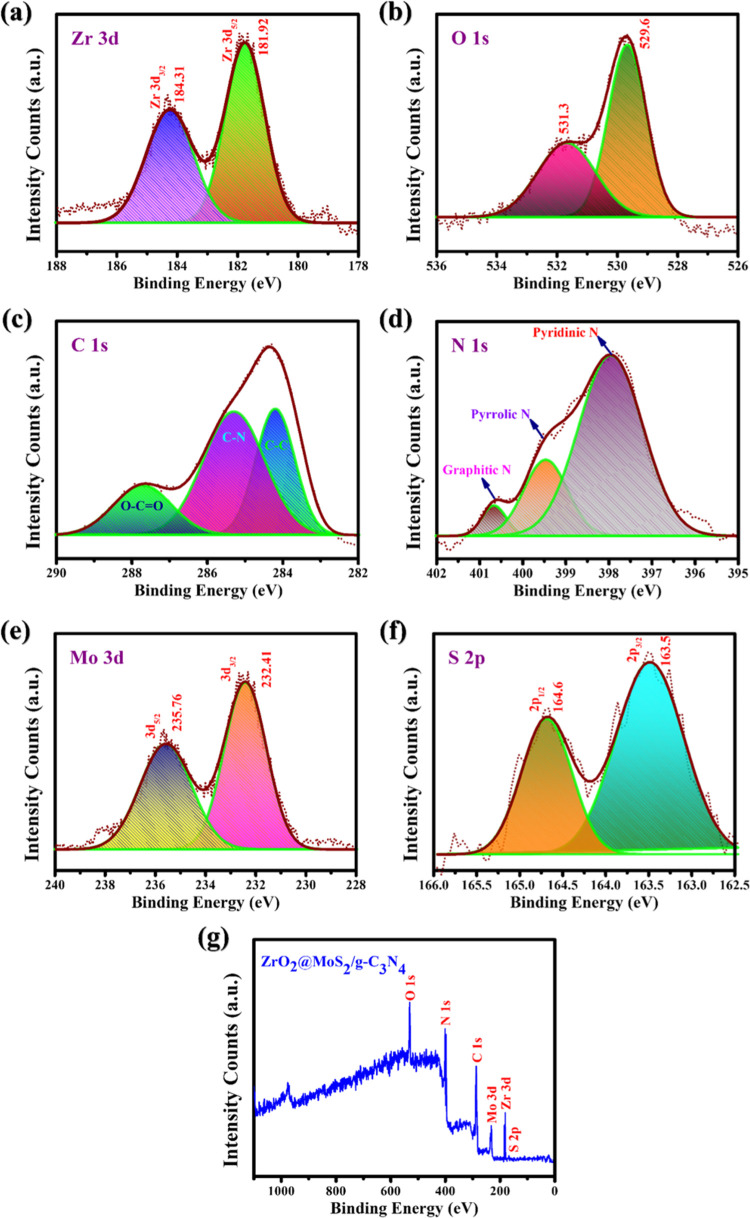
XPS spectra of the ZrO_2_@MoS_2_/g-C_3_N_4_ nanocomposite. (a) Zr 3d, (b) O 1s, (c) C 1s, (d) N 1s, (e) Mo 3d, and (f) S 2p (g) survey profiles and panels.

The XPS analysis presented in Figure 6 reveals changes in the chemical composition and surface electronic interaction of Zr, O, C, N, Mo, and S components in the ZrO_2_@MoS_2_/g-C_3_N_4_ nanocomposite. As shown in Figure 6a, the Zr 3d_5/2_ and Zr 3d_3/2_ diffraction peaks in the bare ZrO_2_ sample appeared to be at 181.9 and 184.3 eV, respectively.^33^ ZrO_2_ nanoparticles were found to have Zr–O–Zr and Zr–O–H bonding, as shown by their O 1s diffraction values of 529.6 and 531.3 eV, respectively, in Figure 6b.^34^ According to Figure 6c, the electronic states of graphite-like sp^2^ (C–C) and sp^3^ (N–C=N) in the ZrO_2_@MoS_2_/g-C_3_N_4_ nanocomposite are denoted by the C 1s deflection patterns at 284.8 and 288.2 eV, respectively, whereas the diffraction patterns at 286.4 eV mimicked a tiny amount of C–O.^35,36^ In the ZrO_2_@MoS_2_/g-C_3_N_4_ nanocomposite, the N 1s diffraction peaks at pyridinic N (398.6 eV), pyrrolic N (399.5 eV), and graphitic N (401.0 eV) correlate to the (C–N=C), (N–(C)_3_), and N–H bondings, respectively, as shown in Figure 6d.^37^ Among the prominent peaks are Mo 3d_3/2_ (232.4 eV) and Mo 3d_5/2_ (235.7 eV), corresponding to the Mo^4+^ and S 1s states in MoS_2_, respectively. As shown in Figure 6e,f, the formation of tiny amounts of Mo oxides with an adsorbed oxygen molecule results in a higher binding energy pattern (235.7 eV), which is apparent. However, as shown by XRD, there is no oxide phase in Mo, which does not affect the photocatalytic activity of the ZrO_2_@MoS_2_/g-C_3_N_4_ nanocomposite.^38^ According to Figure 6g, the overall element profile in the ZrO_2_@MoS_2_/g-C_3_N_4_ nanocomposite is as follows. A considerable amount of interaction between the MoS_2_/g-C_3_N_4_ and the MoS_2_ would result in this instance. ZrO_2_ also affected the interfacial contact and charge transport of the material, resulting in the C–N pattern in g-C_3_N_4_ shifting to a higher binding energy in composites such as MoS_2_/g-C_3_N_4_ and ZrO_2_@MoS_2_/g-C_3_N_4_. ZrO_2_ is morphologically restricted, and the addition of MoS_2_/g-C_3_N_4_ prevents the object’s interface communication and charge transfer, altering XPS peaks in the Zr 3d and O 1s spectra. The Zr/O, C/N, and Mo/S ratios of the ZrO_2_@MoS_2_/g-C_3_N_4_ nanocomposite are shown in the atomic ratio analysis in Table 1.^39^

**Table 1:** Atomic Ratios of Zr, O, C, N, Mo, and S Derived from the XPS Data of ZrO_2_@MoS_2_/g-C_3_N_4_

element	Zr [atom %]	O [atom %]	C [atom %]	N [atom %]	Mo [atom %]	S [atom %]
ZrO_2_@MoS_2_/g-C_3_N_4_	3.21	36.12	29.13	31.17	0.13	0.24

**Figure 7:**
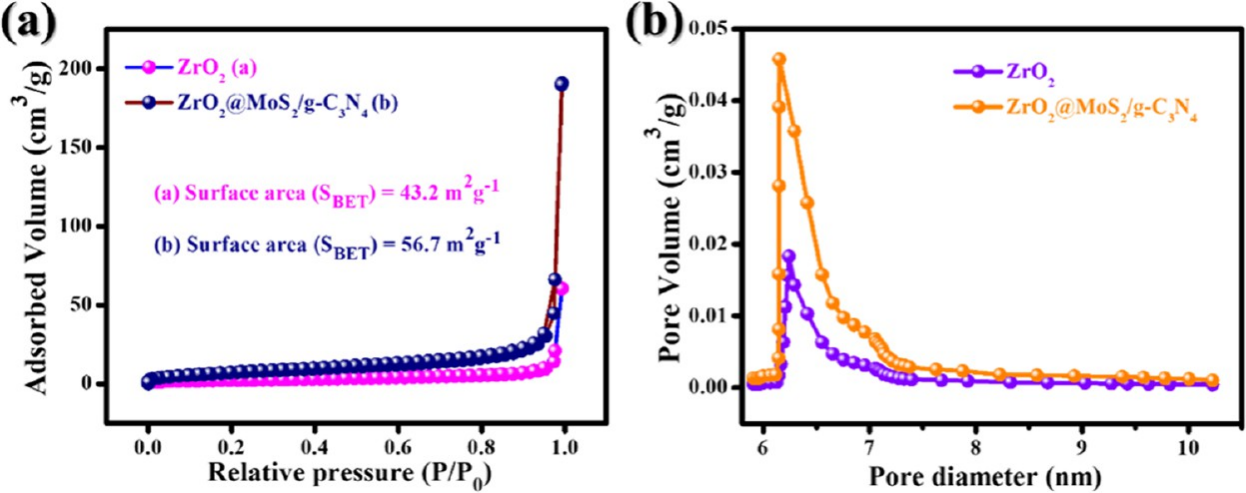
(a) N_2_ adsorption–desorption isotherms and (b) pore size distribution curves of ZrO_2_ and ZrO_2_@MoS_2_/g-C_3_N_4_ nanocomposites.

It is seen that the surface area (*S*_BET_) of the photocatalyst is essential for determining the number of active sites and the charge-carrier transit distance. Figure 7a,b depicts the isotopes of the nitrogen absorption–desorption and hole size distribution curves of ZrO_2_ and ZrO_2_@MoS_2_/g-C_3_N_4_. The ZrO_2_ sample exhibits the H_2_-type hysteresis loop with type IV isotherms (*P*/*P*_0_ = 0.85–1.0), which confirms the constant size of the nanoparticles with a mesopore phase morphology,^40^ As shown in Figure 7a, the ZrO_2_@MoS_2_/g-C_3_N_4_ nanocomposite shows type IV and H_2_ hysteresis loops. Two specimens demonstrated the presence of mesopores. According to the BET approach, the S_BET_ values of ZrO_2_ and ZrO_2_@MoS_2_/g-C_3_N_4_ are 43.2 and 56.7 m^2^ g^–1^, respectively. The surface area of the ZrO_2_@MoS_2_/g-C_3_N_4_ nanocomposite is higher than that of ZrO_2_. To enhance the photocatalytic activity, the wide surface area of the sample provides a sizeable active site for light absorption, contaminant absorption, and photodegradation.^41^ The pore size distribution of ZrO_2_ nanoparticles is 15 nm, while that of the composite ZrO_2_@MoS_2_/g-C_3_N_4_ is in the 10 nm range, with larger mesopores (Figure 7b). The porous MoS_2_/g-C_3_N_4_ not only served as a supporter but also prevented the ZrO_2_@MoS_2_/g-C_3_N_4_ nanocomposite from reaggregating as a result of the improved more extensive nitrogen acceptance and *S*_BET_ of 56.7 m^2^ g^–1^, which was beneficial because the composite sample’s surface will absorb more visible light, thereby increasing the photocatalytic efficiency by providing more active sites.^42^

**Figure 8:**
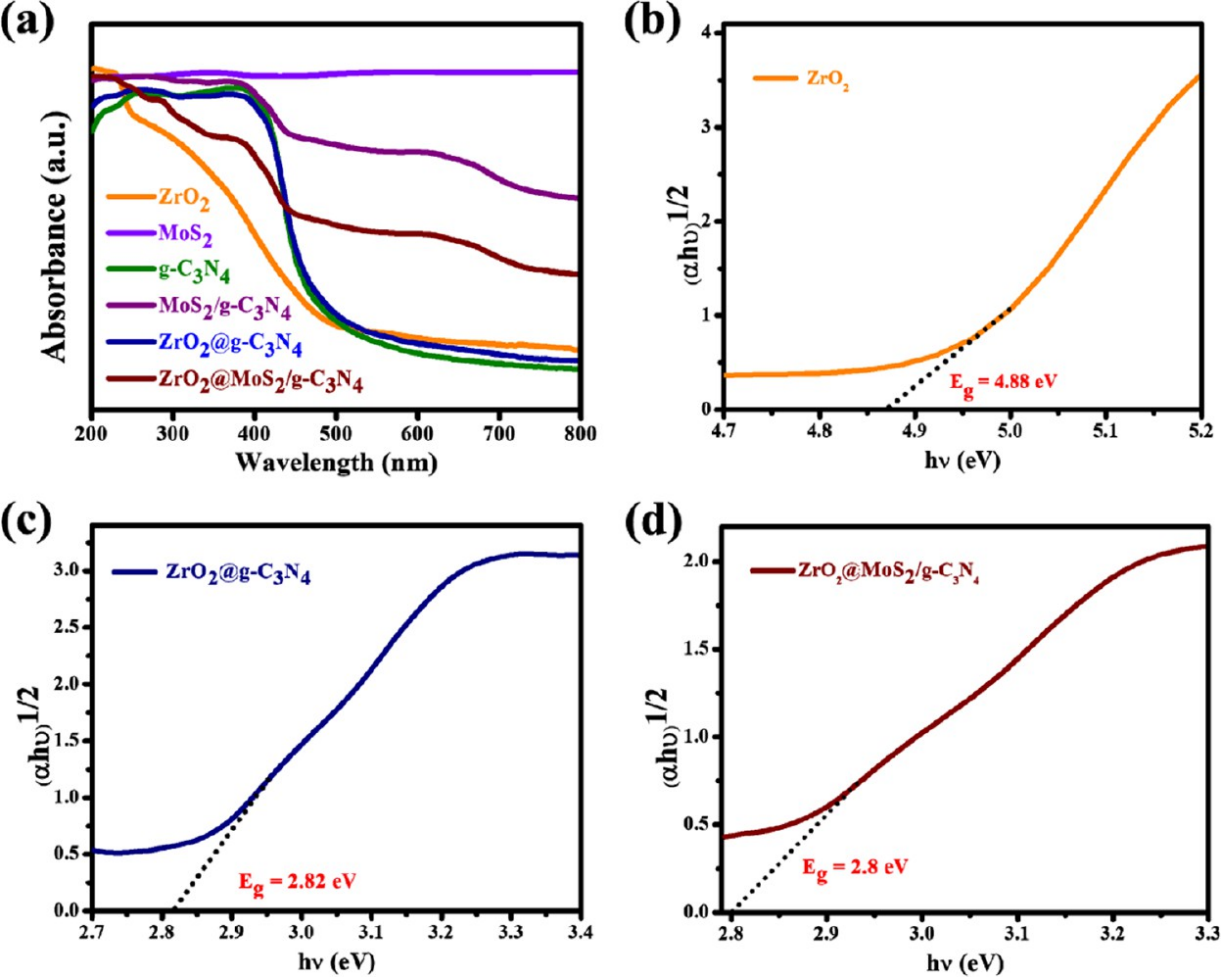
(a) UV–vis DRS spectra of ZrO_2_, g-C_3_N_4_, MoS_2_, MoS_2_/g-C_3_N_4_, ZrO_2_@g-C_3_N_4_, and ZrO_2_@MoS_2_/g-C_3_N_4_ nanocomposites, and (b–d) corresponding Tauc plots of ZrO_2_, ZrO_2_@g-C_3_N_4_, and ZrO_2_@MoS_2_/g-C_3_N_4_ nanocomposites.

To investigate the optical absorbance nature of ZrO_2_, g-C_3_N_4_, MoS_2_, MoS_2_/g-C_3_N_4_, ZrO_2_@g-C_3_N_4_, and the nanocomposite ZrO_2_@MoS_2_/g-C_3_N_4_, UV–vis diffuse reflectance spectra were obtained (Figure 8a). In the visible range, g-C_3_N_4_ has an absorption onset in the visible 460 nm, while bare ZrO_2_ is absorbed in the UV radiation wavelength range (absorption starts at 275 nm). As expected, the absorption onset of ZrO_2_@MoS_2_/g-C_3_N_4_ occurs between its constituents at ∼490 nm. Additionally, the band gap of ZrO_2_ (4.88 eV) is in agreement with the reported literature.^43,44^ The addition of MoS_2_/g-C_3_N_4_ resulted in a modest modification in the Tauc plots, as illustrated in Figure 8b–d. As a result of the shift in absorbance edges for the nanocomposite structure, the band gaps for ZrO_2_@g-C_3_N_4_ and ZrO_2_@MoS_2_/g-C_3_N_4_ are 2.82 and 2.8 eV, respectively. The fact that such a modest change in band spacing supports the g-C_3_N_4_ and MoS_2_ sheet production with tight interface contact indicates the presence of ZrO_2_ nanoparticles and nanocomposite synthesis. Theoretically, the nanosize composite has a larger contact interface area, which allows for a faster charging carrier transit and, as a result, suppresses charge recombination, resulting in increased photocatalytic degradation efficiency.^45^

**Figure 9:**
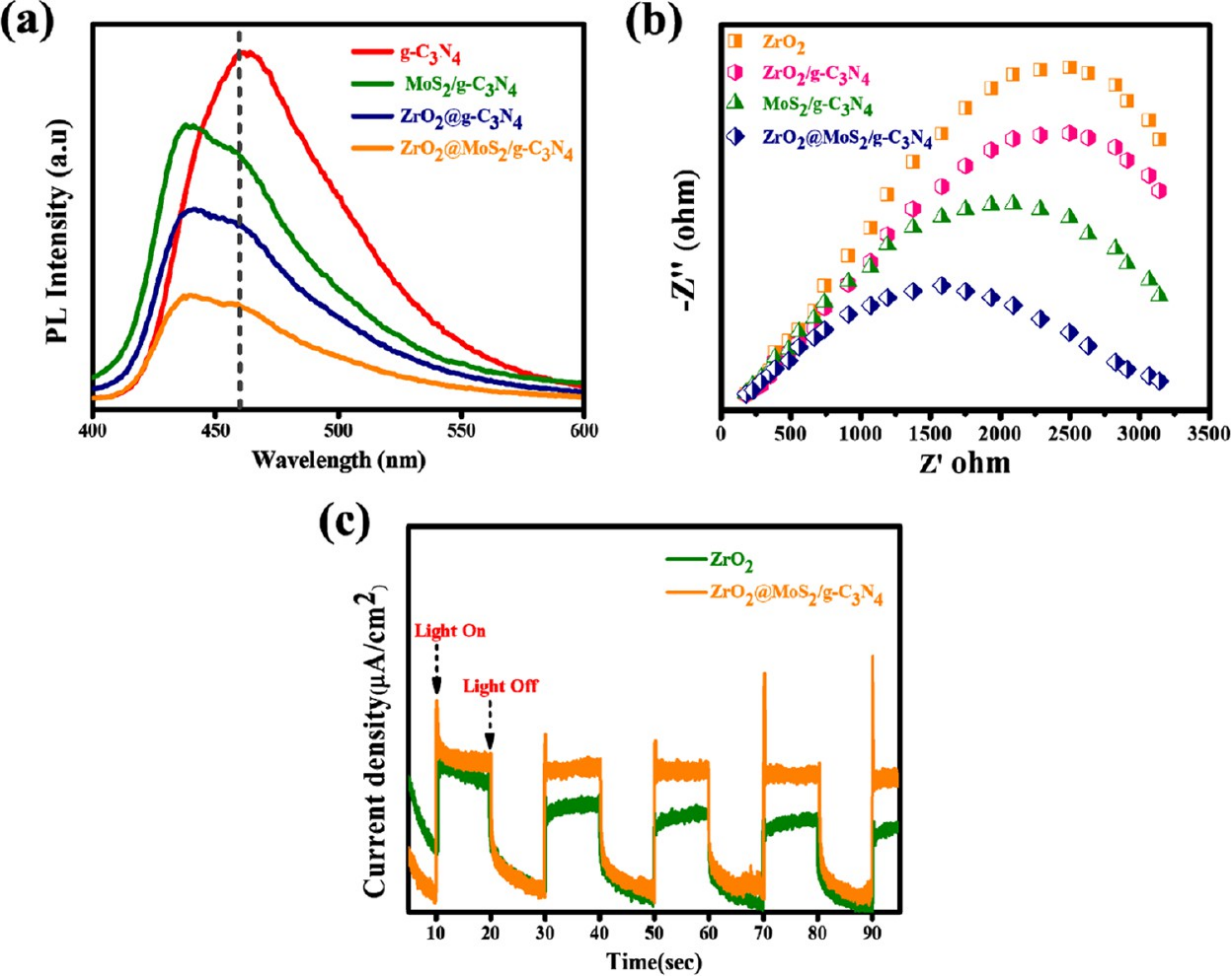
(a) PL spectra of g-C_3_N_4_, MoS_2_/g-C_3_N_4_, ZrO_2_@g-C_3_N_4,_ and ZrO_2_@MoS_2_/g-C_3_N_4_. (b) EIS Nyquist plots of ZrO_2_, ZrO_2_@g-C_3_N_4_, MoS_2_/g-C_3_N_4_, and ZrO_2_@MoS_2_/g-C_3_N_4_ nanocomposites. (c) Transient photocurrent response of ZrO_2_ and ZrO_2_@MoS_2_/g-C_3_N_4_ nanocomposites.

The photoluminescence spectra were primarily conducted to evaluate the charge transfer and separation behaviors to analyze the starting point of the enhanced photocatalytic activity. Figure 9a shows that, under 325 nm laser stimulation, pure g-C_3_N_4_ exhibits an exceptionally bright photoluminescence pattern with the emission wavelength centered at about 460 nm, which can be attributed to its fast charge recombination. In the presence of ZrO_2_@g-C_3_N_4_ and MoS_2_/g-C_3_N_4_, the emission peak of the mixture is slightly lowered as a result of poor interactions between the constituents in the mechanical mix.^46^ The EIS Nyquist plot provides an additional context for exploring spatial charge-transfer assets. The lower the charge-transfer resistance in the broad spectrum, the smaller the semicircle diameter of the EIS Nyquist plot as the spectrum narrows. Following Figure 9b, the relative arc diameters of the samples can be organized in the following ways: ZrO_2_ > ZrO_2_@g-C_3_N_4_ > MoS_2_/g-C_3_N_4_ > ZrO_2_@MoS_2_/g-C_3_N_4_, indicating that the nanocomposite interaction between ZrO_2_ and MoS_2_/g-C_3_N_4_ will result in the realization of a rapid and effective spatial charge separation process. The above analysis reveals that a more significant number of electrons and holes are used in the photocatalytic process in the ZrO_2_@MoS_2_/g-C_3_N_4_ nanocomposite-based reaction compared to the conventional reaction. As illustrated in Figure 9c, transient photocurrent measurements of ZrO_2_@MoS_2_/g-C_3_N_4_ reveal a higher photocurrent density than that of pure ZrO_2_, indicating a very effective separation of photoexcited charges (electron–hole pairs) and restriction of their recombination. Several repeats of cycles display a similar photocurrent reaction. Significantly, it can be seen that the samples have high photostability.^47^

**Figure 10:**
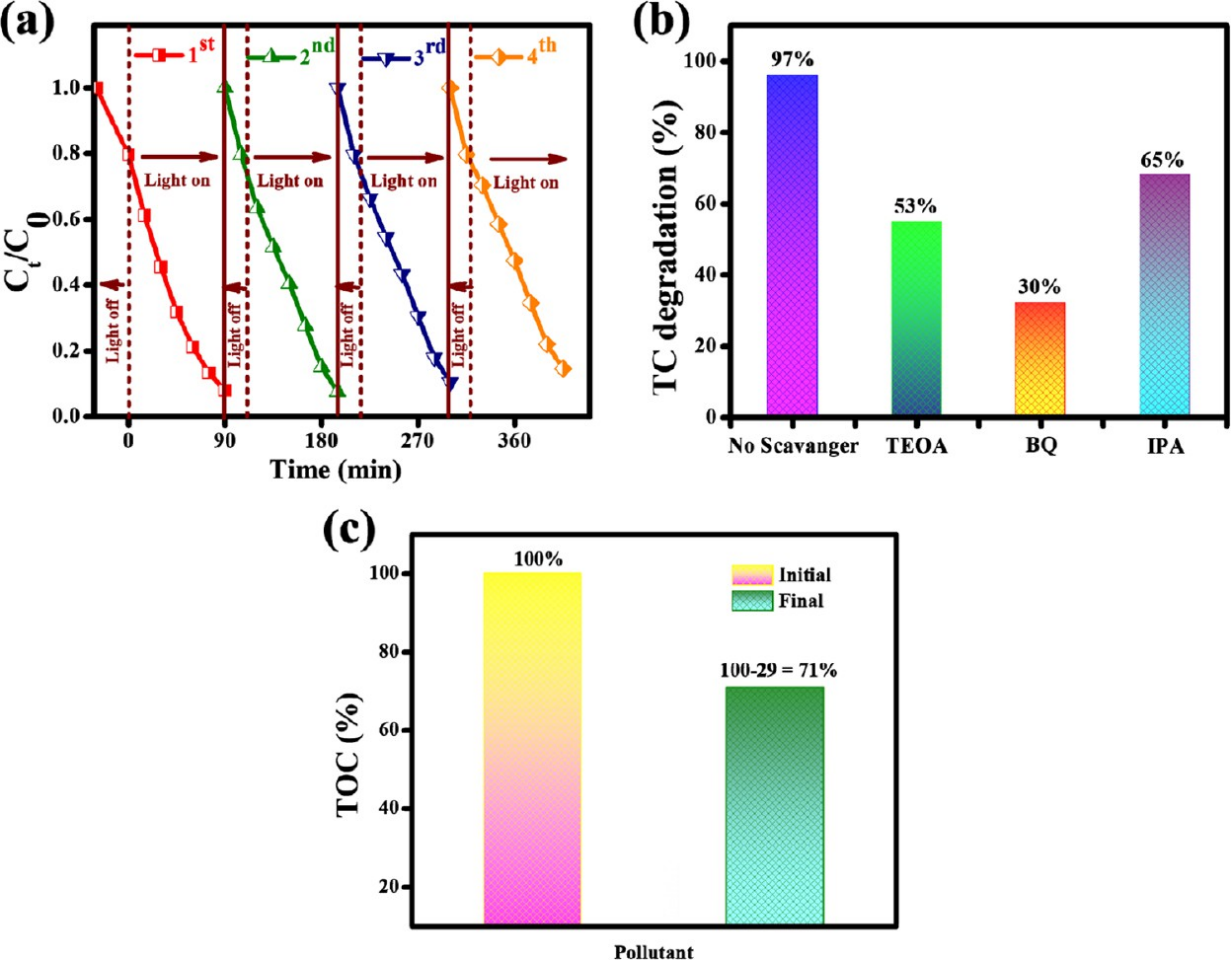
(a) Recycle efficiency of the ZrO_2_@MoS_2_/g-C_3_N_4_ photocatalyst for four cycles. (b) Percentage of the photocatalytic degradation of TC with ZrO_2_@MoS_2_/g-C_3_N_4_ in the presence of different scavengers (TC conc.: 20 mg/L; ZrO_2_@MoS_2_/g-C_3_N_4_ 50 mg; TEOA: (1.6 × 10^–4^ mol L^–1^) in 100 mL/BQ – (4.0 × 10^–4^ mol L^–1^) 100 mL/IPA – (1.0 × 10^–1^ mol L^–1^) in 100 mL, irradiation time: 90 min), (c) TOC mineralization efficiency for TC of the ZrO_2_@MoS_2_/g-C_3_N_4_ catalyst.

The recycle productivity of ZrO_2_@MoS_2_/g-C_3_N_4_ was examined by recovering the photocatalyst, and the results are given in Figure 10a. The degradation percentages were 94.8 and 92%, respectively, when compared to the first two recycling cycles. Photocatalytic efficiencies decreased to 91 and 90.1% for the third and fourth recycling cycles, respectively. Until the photocatalyst was used in the fourth cycle, there was little change in the degradation percentage. The absorption of the TC solution on the surface of the photocatalyst may be responsible for the slight divergence in the photodegradation during the recycling experiment. The photocatalytic process is carried out in various sacrificial agents using a ZrO_2_@MoS_2_/g-C_3_N_4_ catalyst to identify the active species that participate in TC degradation and remove specific reactive species. Continuous radical scavenging studies are being performed to evaluate the mechanism of ZrO_2_@MoS_2_/g-C_3_N_4_ for TC degradation. In the tests, BQ, triethanolamine (TEOA), and isopropanol (IPA) were used as ^•^O_2_^–^, h^+^, and ^•^OH scavengers, respectively. As illustrated in Figure 10b, the TC degradation efficiency of the ZrO_2_@MoS_2_/g-C_3_N_4_ nanocomposite is significantly suppressed in the presence of BQ (30%), which shows the primary quencher of hydroxyl (e^–^) species in the degradation reaction, compared to a hole (h^+^) and electron (e^–^) scavenger. From the absorbed oxygen molecule, photogenerated electrons (e^–^) form ^•^O_2_^–^ radicals, and ^•^OH species form from the hole (h^+^) in the aqueous solution. In the optimal ZrO_2_@MoS_2_/g-C_3_N_4_ nanocomposite [TC = 20 mg/L at pH 3], the TOC technique was used to evaluate TC mineralization. Figure 10c shows that the TC mineralization efficiency was 71%, and the photocatalytic efficiency was 94.7% under 90 min of radiation. These results indicated that the ZrO_2_@MoS_2_/g-C_3_N_4_ nanocomposite presents the highest mineralization ability in the TC degradation process.^48^

**Figure 11:**
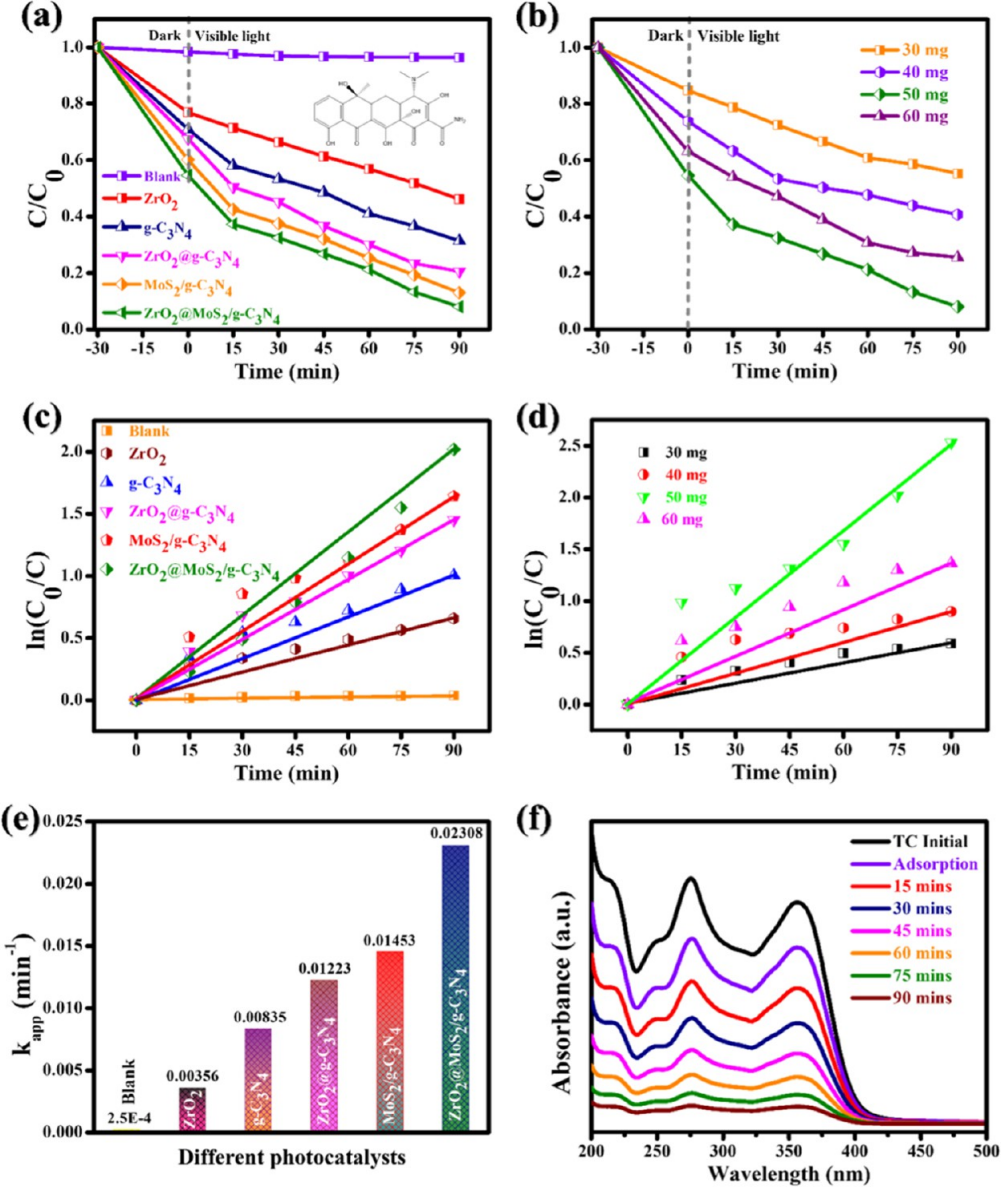
Photocatalytic degradation of TC under different conditions: (a) different catalysts (catalysts: 50 mg; TC: 20 mg/L), (b) different dosages of ZrO_2_@MoS_2_/g-C_3_N_4_ [TC conc.: 20 mg/L], (c) pseudo-first-order kinetic plots of ln(*C*_0_/*C*) vs time for different catalysts and (d) different dosages of ZrO_2_@MoS_2_/g-C_3_N_4_, (e) kinetic constants of different catalysts (TC: 20 mg/L; catalyst dose = 50 mg), and (f) absorbance spectrum (TC: 20 mg/L; catalyst dose = 50 mg).

**Figure 12:**
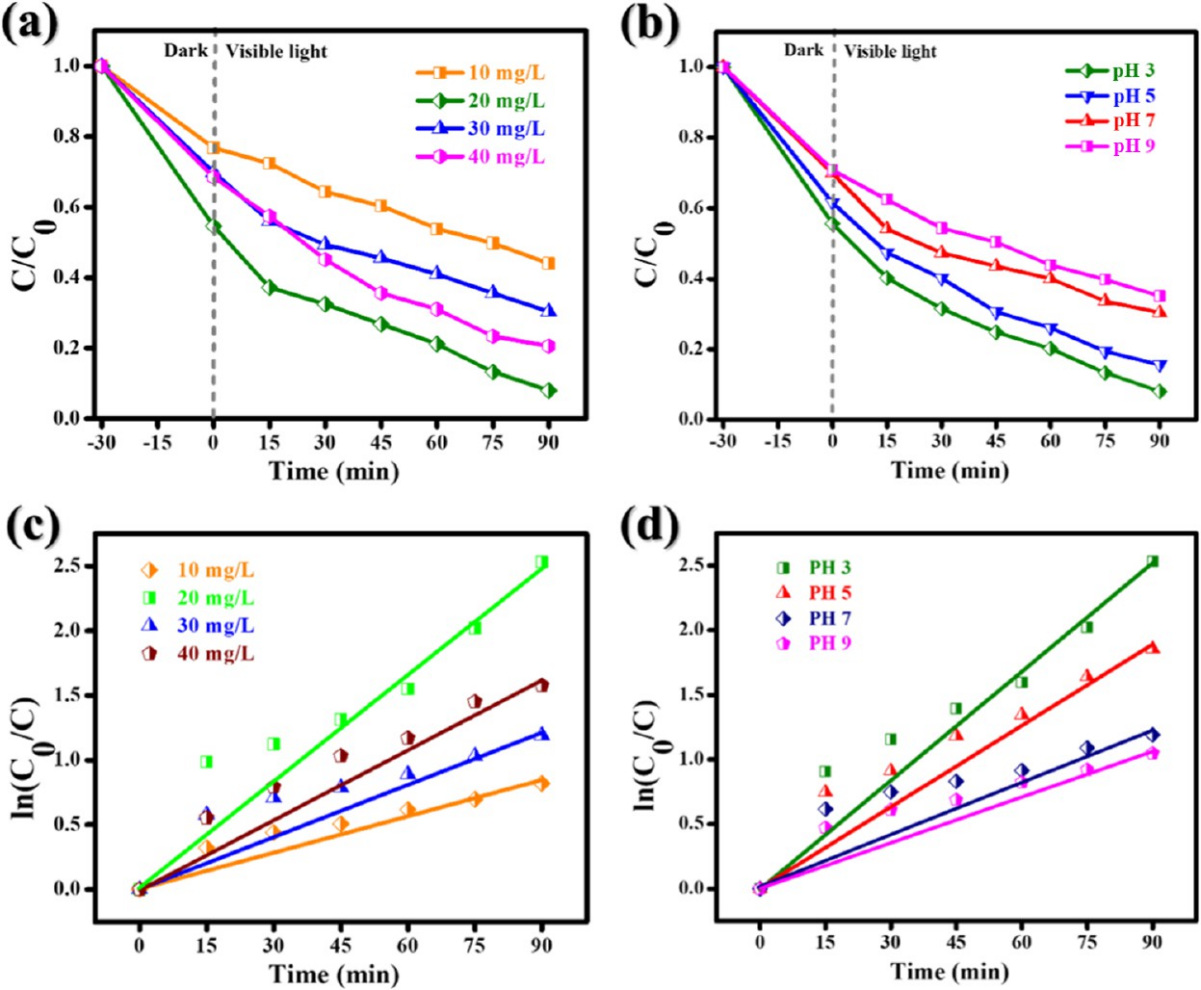
Photocatalytic degradation of TC under different concentrations: (a) different TC concentrations (catalyst dose = 50 mg), (b) different pH conditions of TC in the presence of ZrO_2_@MoS_2_/g-C_3_N_4_, (c) first-order kinetic plots of ln(*C*_0_/*C*) vs time for different concentrations, and (d) different pH of TC in the presence of ZrO_2_@MoS_2_/g-C_3_N_4_ (TC: 20 mg/L; catalyst dose = 50 mg).

The photocatalytic degradation activity of all prepared photocatalysts is determined by monitoring the decomposition of TC in an aquatic solution under visible-light illumination, as illustrated in Figure 11. The primary blank test demonstrated that no degradation could be identified in the absence of light, indicating that the photocatalyst was the main reason for the degradation. The minor decrease in the TC content of ∼4% is due to adsorption in the blank condition. After 90 min, the photocatalytic activity of ZrO_2_@MoS_2_/g-C_3_N_4_ is the highest among them, and the degradation rate of TC when using this compound reaches 94.8%. When compared to other photocatalysts, the composite material ZrO_2_@MoS_2_/g-C_3_N_4_ was more efficient than ZrO_2_, g-C_3_N_4_, ZrO_2_@ g-C_3_N_4_, and MoS_2_/g-C_3_N_4_. The percentages of TC degradation for photocatalysts ZrO_2_, g-C_3_N_4_, ZrO_2_@g-C_3_N_4_, MoS_2_/g-C_3_N_4_, and ZrO_2_@MoS_2_/g-C_3_N_4_ are 41.9, 68.6, 79.3, 86.9, and 94.8% under visible-light irradiation in 90 min, respectively, as shown in Figure 11a. To determine the optimal dose of ZrO_2_@MoS_2_/g-C_3_N_4_ in TC degradation, various weight ratios, including 30, 40, 50, and 60 mg of photocatalyst, were used for photocatalytic degradation and examined under comparable test circumstances, as shown in Figure 11b. When comparing the various weight ratios of ZrO_2_@MoS_2_/g-C_3_N_4_, the photodegradation efficiencies of the various weight ratios were 44.6, 59.4, 94.8, and 74.4%, corresponding to the 30, 40, 50, and 60 mg weight ratios. Increasing the photocatalyst volume enhances the absorption, but increased photocatalyst density accelerates the TC degradation.^49^ Under these test conditions, it is reported that 50 mg/100 mL of photocatalyst is the most efficient and optimal for the effective degradation of TC when exposed to visible-light irradiation for 90 min. With an increase in the photocatalyst concentration, the TC degradation rate increases to 50 mg/100 mL, and an increase in the photocatalyst concentration further reduces the degradation rate. When photocatalyst particles are present in quantities more than 60 mg/100 mL (50 mg/100 mL), the dispersion of light by the particles slows down the rate of degradation.^50^ The ZrO_2_@MoS_2_/g-C_3_N_4_ nanocomposite dose and photocatalyst concentrations in Figure 11c,d show the photodegradation kinetics of TC. The pseudo-first-order rate constants are 0.0035, 0.0083, 0.0122, 0.0145, and 0.0230 min^–1^ (in Figure 11e) for ZrO_2_, g-C_3_N_4_, ZrO_2_@g-C_3_N_4_, MoS_2_/g-C_3_N_4_, and ZrO_2_@MoS_2_/g-C_3_N_4_, respectively. The photocatalyst weights of 30, 40, 50, and 60 mg are represented by the kinetic rate constant values of 0.0060, 0.0084, 0.0236, and 0.0140 min^–1^, respectively. Photocatalytic degradation occurs best under conditions of visible-light illumination, as measured by kinetic rate constants. The determined values are 0.0230 min^–1^ for the composite material and 0.0236 min^–1^ for 50 mg of the photocatalyst. According to Figure 11f, the absorption band’s intensity reduces in the presence of the ZrO_2_@MoS_2_/g-C_3_N_4_ catalyst with the intensity of the absorption band decreasing in proportion to the time of visible-light irradiation. The UV absorption at 357 nm decreases over time due to the decay of TC.^51^ The effect of different TC concentrations (10, 20, 30, and 40 mg/L TC) on the photocatalytic degradation of TC was studied to establish the optimal state of the catalyst utilized. TC degradation increases from 55.9, 93.8, 69.6, and 79.3%, respectively. Because of the homogeneity of the catalyst and the challenge of light that enters the catalyst surface, the photocatalytic degradation reduces as the TC concentration increases to 40 mg/L. Figure 12a illustrates this phenomenon.^52^ When a starting TC concentration of 20 mg/L was used, the photodegradation of TC by ZrO_2_@MoS_2_/g-C_3_N_4_ was studied at different pH values of 3, 5, 7, and 9, and the degradation percentage is shown in Figure 12b. It takes 90 min of visible-light illumination to significantly reduce the TC degradation percentages to 93.8, 88.3, 70.1, and 60.5% at pH levels of 3, 5, 7, and 9, respectively. The best performance is for TC degradation at pH levels 3 and 5. According to previous reports, TC is amphoteric, with p*K*_a_ values of 3.4, 7.7, 9.8, and 12. It is primarily in the neutralized form TC^0^ from pH 3.4–7.7 and the deprotonated form TC^–^ at pH 7.7–12. The charge intensity of TC increased with pH increasing, resulting in a more intense species attack against molecules.^53^ The kinetic curves in Figure 12c,d were used to derive the rate constants for various TC concentrations. Parameters 10, 20, 30, and 40 mg/L TC and pH 3, 5, 7, and 9 are mimicked by pseudo-first-order rate constants of 0.0191, 0.0263, 0.0131, and 0.0179 min^–1^. The maximal operating rates for maximum decay were 0.0263 and 0.0238 min^–1^ at 20 mg/L and pH 3, respectively. Table 2 lists some statistics to compare with other early reports on photodegradation of TC by composite photocatalysts.

**Table 2:** Comparison of Photocatalytic Efficiencies of TC Degradation for Different Photocatalysts

photocatalyst	*C*_catalyst_ dose (mg)	*C*_TC_ conc. (mg/L)	light source (Xe lamp, W)	kinetic constant (min^–1^)	degradation (%) (time)	ref
Fe_3_O_4_@BiOCl/BiVO_4_	50	20 (100 mL)	300 (λ > 420 nm)	0.0263	87 (90 min)	(2)
SiO_2_-Fe_2_O_3_@TiO_2_	10	10 (50 mL)	300 (λ > 420 nm)		80 (80 min)	(4)
g-C_3_N_4_/ZrO_2_	2	10 (5 mL)	300 (λ > 420 nm)	0.0474	90.6 (60 min)	(16)
MoS_2_/B-rGO	20	40 (100 mL)	300 (λ > 420 nm)	0.0203	85.2 (90 min)	(20)
Au/Pt/g-C_3_N_4_	100	20 (100 mL)	500 (λ > 400 nm)	0.4286	93 (180 min)	(33)
WO_3_/g-C_3_N_4_	50	25 (100 mL)	300 (λ > 420 nm)	0.0120	70 (120 min)	(60)
Sn_3_O_4_/g-C_3_N_4_	50	10 (100 mL)	500 (λ > 420 nm)	0.0108	72.2 (120 min)	(61)
g-C_3_N_4_/Nb_2_O_5_	100	20 (100 mL)	250 (λ > 420 nm)	0.0096	76.2 (150 min)	(62)
Bi/α-Bi_2_O_3_/g-C_3_N_4_	50	10 (50 mL)	300 (λ > 400 nm)	0.0122	91.2 (180 min)	(63)
BiOI/g-C_3_N_4_/CeO_2_	50	20 (30 mL)	300 (λ > 420 nm)	0.0205	91.6 (120 min)	(64)
ZrO_2_@MoS_2_/g-C_3_N_4_	50	20 (100 mL)	300 (λ > 420 nm)	0.0230	94.8 (90 min)	this work

**Figure 13:**
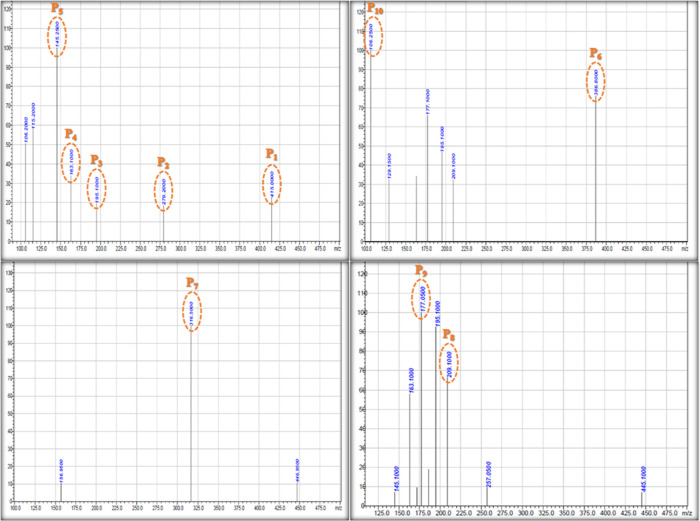
LC–MS spectra of the TC degradation intermediate products in the presence of the ZrO_2_@MoS_2_/g-C_3_N_4_ nanocomposite photocatalyst after 90 min of irradiation time.

**Figure 14:**
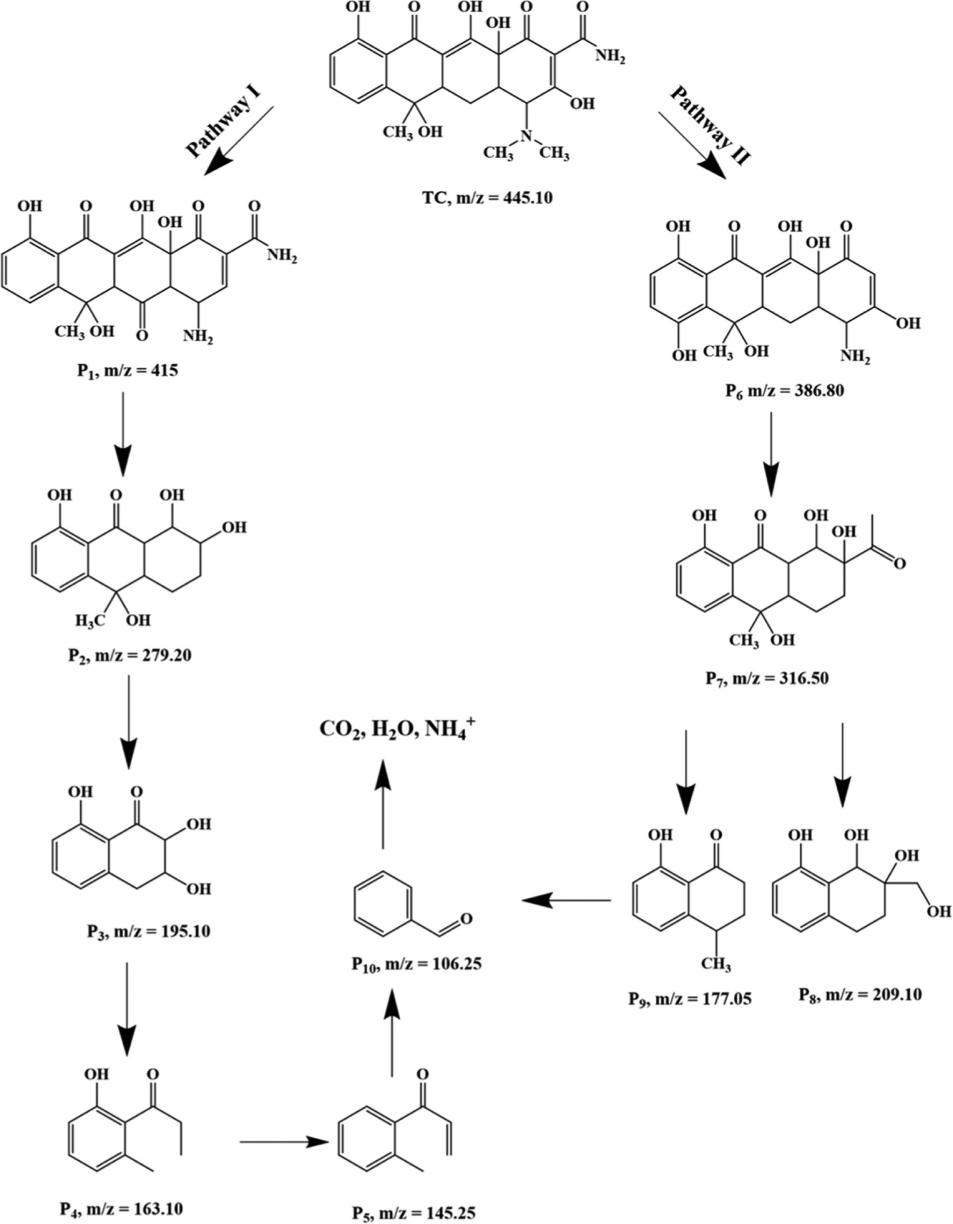
Proposed possible degradation pathway and primary intermediate photoproducts of TC in the ZrO_2_@MoS_2_/g-C_3_N_4_ nanocomposite with the combined photocatalytic system.

The presence of the ZrO_2_@MoS_2_/g-C_3_N_4_ nanocomposite degraded the TC in around 90 min, as analyzed by liquid chromatography–mass spectrometry (LC–MS). LC–MS analysis clarified the photodegradation pathway of TC. The uniform mass spectrum obtained after 90 min of reaction with TCs and the resulting mass spectra are shown in Figure 13. Figure 13 shows that TC was reduced to 10 primary photointermediate pathways (I and II), which are denoted as P_1_–P_10_ in the direction of the maintenance period. Additionally, based on the positive m/z ratios discovered and the findings of previous studies, the structural and chemical formulae of restricted byproducts were established, as shown in Figure 13. In addition, a possible photodegradation route was proposed, as revealed in Figure 14. The N-demethylation of TC resulted in the production of Pathway and an intermediate of P_1_ with an *m*/*z* of 415 (*m* + 1)^+^. Further photodegradation led to product P_1_ led to product P_2_ (*m*/*z* 279.20) (*m* + 2)^+^, which was formed due to the loss of the formamide group and subsequent oxidation, resulting in the construction of the resulting hydroxyl structure. Due to the low C–N binding energy, the carboatomic ring disintegrates. In other words, they can be attributed to the development of product P_3_ (*m*/*z* 195.10) (*m* + 1)^+^. Meanwhile, due to the low binding energy of the carbonyl and hydroxyl groups, the carbonyl and hydroxyl groups were removed. As shown in Figure 14, active groups were removed further, resulting in product P_4_ (*m*/*z* 163.10) (*m* + 1)^+^, the end product. Product P_5_ (*m*/*z* 145.25) (*m* – 1)^+^ was formed due to the dissociation of the oxidation of the hydroxyl group in product P_4_. In pathway II, the radicals may initially react with the C=C of TC, resulting in a composite fabrication with an *m*/*z* value of P_6_ 386.80 (*m* – 2)^+^. In one photodegradation pathway, additional confronting of free radicals results in the cleavage of the aromatic ring at C=C, which produces the combinations with m/z values of P_7_ 316.50, P_8_ 209.10 (*m* + 1)^+^, P_9_ 177.10 (*m* + 1)^+^, and P_10_ 106.25. Finally, the fragments of this photointermediate were degraded to form CO_2_, NH_4_^+^, and H_2_O.^54−56^

**Figure 15:**
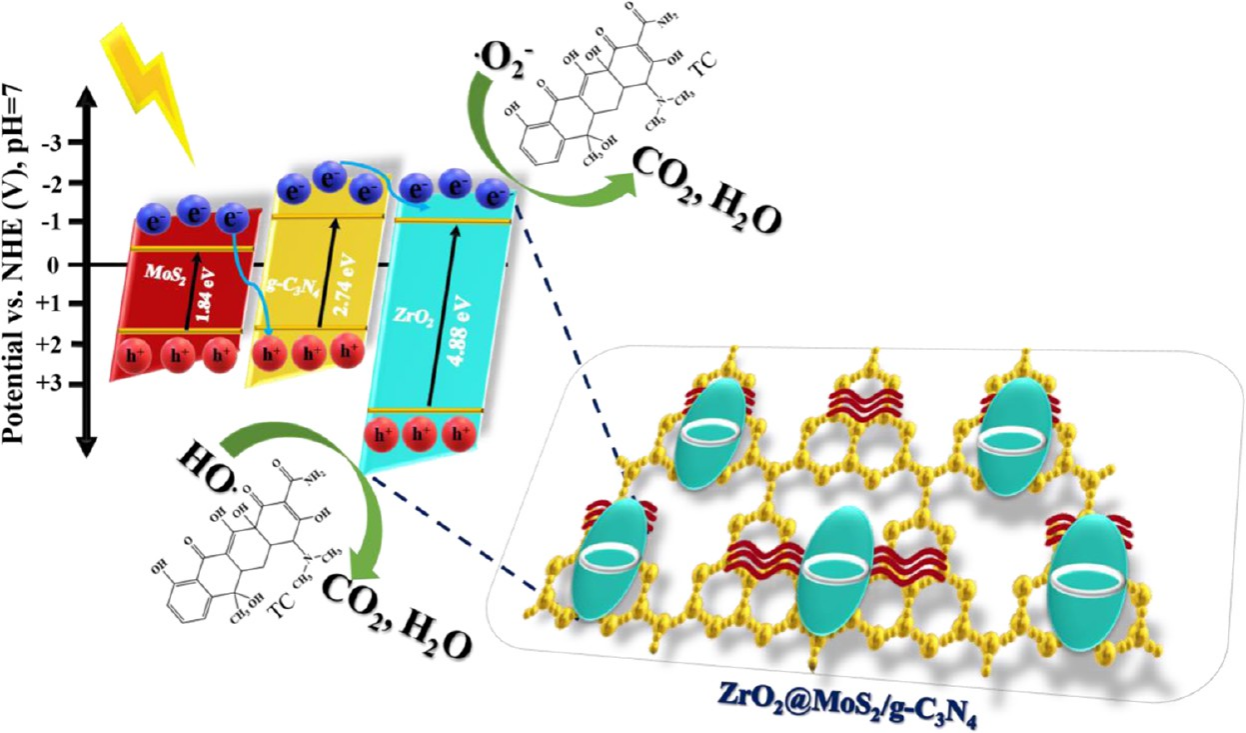
Proposed possible photocatalytic degradation mechanism of TC in the ZrO_2_@MoS_2_/g-C_3_N_4_ nanocomposite.

In a subsequent investigation, the variation of radical species in the photocatalytic mechanism of photodegradation of TC on the catalyst occurring is shown in Figure 15. However, the radical trapping experiments discussed above proved the existence of the element ^•^O_2_^–^ in the first place. As a result, ^•^OH does not participate as an active species. Due to the high probability of O_2_/^•^O_2_^–^, the CBM values of ZrO_2_ and g-C_3_N_4_ are greater than those of O_2_. Therefore, O_2_ can condense to ^•^O_2_^–^, and the TC is impaired due to the substantial reduction of ^•^O_2_^–^ in the atmosphere. Because of the photoinduced VB to CB electron transition in MoS_2_/g-C_3_N_4_, holes appear in the VBM of semiconductors in the ZrO_2_@MoS_2_/g-C_3_N_4_ system when exposed to visible light. As a result of the change in the CBM capacity, the photoinduced electrons on the surface of MoS_2_/g-C_3_N_4_ migrate to the CB of ZrO_2_, but the holes on the surface of MoS_2_/g-C_3_N_4_ remain on the surface as a result of the change in the CBM capacity.^57,58^ Specifically, when it comes to ZrO_2_, the restricted band energy is high, and visible light is insufficient to attract electrons from their current valence band to their current conduction band. The absorption of visible light by g-C_3_N_4_ allows it to produce the π–π* transition and transfer the excited-state electrons from the VB to the CB. Therefore, electrons transferred from g-C_3_N_4_ to the ZrO_2_ conduction band will not be a barrier to the VB when they enter the bar because it is either a type II charge transfer or a type I charge transfer. The scheme can effectively develop the charge separation of photoinduced electron–hole pairs, thereby significantly reducing the chance of e^–^/h^+^ pair recombination.^59^ The ZrO_2_@MoS_2_/g-C_3_N_4_ nanocomposite exhibits a superior photocatalytic activity because the scheme can effectively develop the charge separation of photoinduced electron–hole pairs, thereby significantly reducing the chance of e^–^/h^+^ pair recombination. In the ZrO_2_@MoS_2_/g-C_3_N_4_ mechanism, the overhead photoluminescence and EIS, electron–hole pair recombination, and photocatalytic efficiency all indicate that the recombination of electron–hole pairs has been curtailed, and the straight oxidation capability of holes has been dramatically increased, resulting in the presence of both h^+^ and ^•^O_2_^–^ as active species in the ZrO_2_@MoS_2_/g-C_3_N_4_ nanocomposite. According to the mechanism (Figure 15), the contact between MoS_2_/g-C_3_N_4_ and ZrO_2_ influences the electron–hole pair separation efficiency. Due to the high quantities of ZrO_2_, the contact size is reduced, boosting the separation efficiency and photocatalytic efficiency. As a result, MoS_2_/g-C_3_N_4_ is the most effective cocatalyst in the ZrO_2_@MoS_2_/g-C_3_N_4_ nanocomposite for photocatalysts in the degradation of TC.
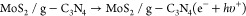
1
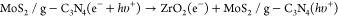
2
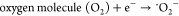
3
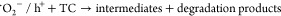
4

## 3 Conclusions

In summary, ZrO_2_ nanoparticles are embedded in a layered MoS_2_/g-C_3_N_4_ composite to form a “particle-embedded-layered” structure via a feasible ultrasonic chemical method. Under visible-light irradiation, the ZrO_2_@MoS_2_/g-C_3_N_4_ nanocomposite has proven to be a viable photocatalyst for tetracycline (TC) degradation. With an apparent kinetic rate constant κ of 0.0230 min^–1^, the photocatalytic degradation efficiency of TC over ZrO_2_@MoS_2_/g-C_3_N_4_ was determined to be around 94.8% in 90 min. The dual charge-transfer channel between the layers of MoS_2_/g-C_3_N_4_ and ZrO_2_ nanoparticles is the reason for the superior photocatalytic activity of ZrO_2_@MoS_2_/g-C_3_N_4_. It promotes the formation of photoinduced charge carriers while also reducing photoinduced charge recombination, resulting in more free charge carriers available to aid photocatalytic reactions via the production of the ^•^O_2_^–^ radical. To account for the removal of the tetracycline from the aqueous solution, the LC–MS measurements for the reaction intermediates, the reaction pathway, and the mechanism were also carried out. As a result, these photocatalysts may also provide feasible and long-term solutions to the environmental issues that antibiotic-polluted effluents cause.

## 4 Experimental Section

### 4.1 Synthesis of ZrO_2_ NPs

About 2.5 mmol of ZrOCl_2_·8H_2_O was ultrasonically scattered in 70 mL of deionized water. The ammonia solution was gradually added with vigorous stirring while maintaining a pH between 10 and 11. The sediments above were transferred to a Teflon-lined stainless-steel autoclave and warmed to 200 °C for 12 h before being left to cool to ambient temperature. The deposits were centrifuged and dehydrated at 80 °C after being rinsed with DI water and ethanol numerous times. The completed products were then calcined for 2 h in static air at 400 °C.

### 4.2 Loading ZrO_2_ NPs onto Layered MoS_2_/g-C_3_N_4_ NSs

**Scheme 1:**
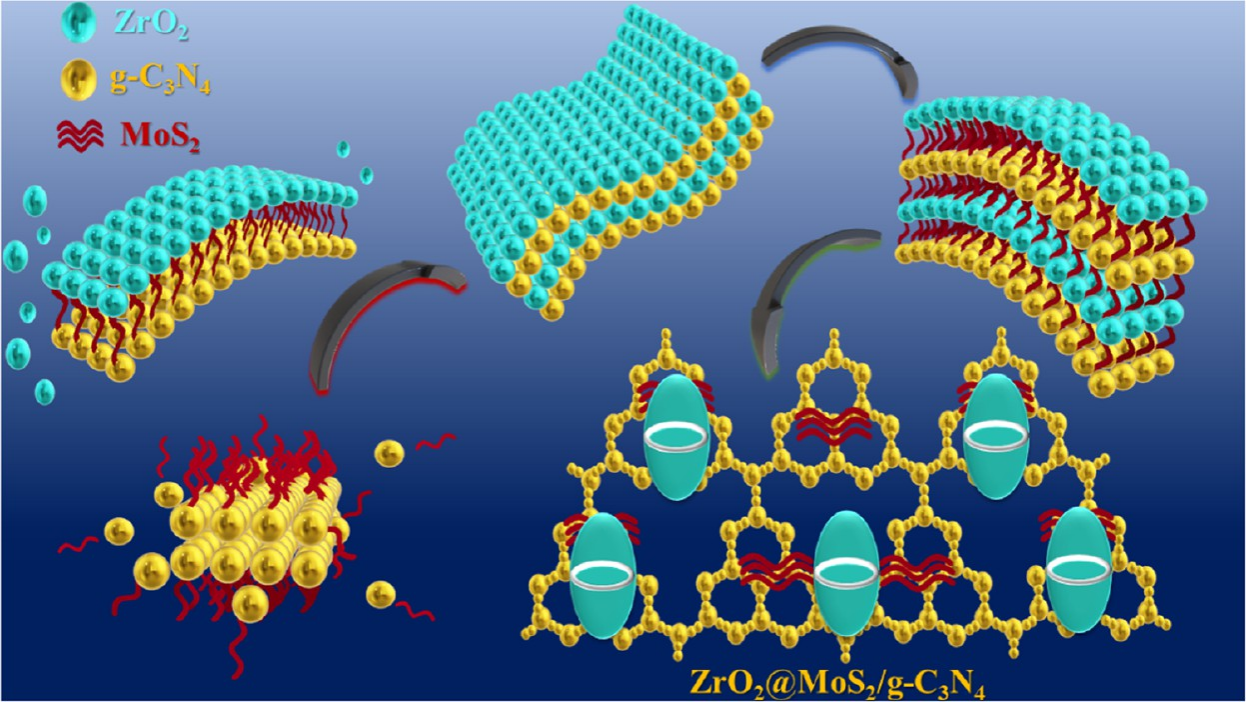
Representation of Feasible Ultrasonic Chemical and Self-Assembly Syntheses of the ZrO_2_@MoS_2_/g-C_3_N_4_ Nanocomposite

Direct heating in melamine at 540 °C for 4 h in a furnace to obtain a graphite-like C_3_N_4_ was accomplished using a muffle furnace. A feasible ultrasonic chemical and self-assembly method (Scheme 1) was used to prepare the ZrO_2_@MoS_2_/g-C_3_N_4_ nanocomposite. A total of 0.5 g of g-C_3_N_4_ powder was generally ultrasonically homogenized in 40 mL of deionized water for 2 h. The sodium molybdate (1 mmol) solution was added to the thiocarbamide (2 mmol) solution, dropped into the above light-yellow suspension, and treated hydrothermally. Following continuous washing and heat-drying, a suitable quantity of stacked MoS_2_/g-C_3_N_4_ and pure ZrO_2_ NPs (0.1 g) was scattered in 40 mL of methanol for 2 h and then violently agitated continuously in a fume cupboard to eliminate the solution. To improve the contact between the MoS_2_/g-C_3_N_4_ layers and the ZrO_2_ matrix, the products were ground and then sintered at 350 °C for 2 h under a static airflow to increase the connection between the layers.

### 4.3 Evaluation of the Photocatalytic Activity and Active Species Capturing Experiments

Using 2 mg of TC and 50 mg of ZrO_2_@MoS_2_/g-C_3_N_4_ catalyst in 100 mL of DI water, the pH of the suspension was calibrated using diluted H_2_SO_4_ and NaOH. Without irradiation, the absorption–desorption equilibrium of the TC pollutant on ZrO_2_@MoS_2_/g-C_3_N_4_ catalysts can be obtained after 30 min of magnetic stirring in the dark. The light source was used as a visible light with a cutoff filter (420 nm ≤ λ ≤ 760 nm) at 300 W Xe. During photocatalytic irradiation, a small aliquot of 4–5 mL of suspension was taken out at 15 min intervals and centrifuged for 10 min at 5000 rpm. Finally, the concentration was calculated using a UV–vis spectrophotometer and the absorption peak value of λ_max_ = 357 nm (TC maximum absorption wavelength).

To detect the active scavenger species, isopropyl alcohol (IPA) (1.0 × 10^–1^ mol L^–1^) was employed as a hydroxyl radical (OH^•^) inhibitor. Benzoquinone (BQ) (4.0 × 10^–4^ mol L^–1^) was utilized to capture the photogenerated superoxide radical (^•^O_2_^–^) and triethanolamine (TEOA) (1.6 × 10^–4^ mol L^–1^) to react with photogenerated holes (h^+^).^65−67^ Preliminary tests were carried out to determine the adequate number of scavengers to use in photocatalytic testing.

### 4.4 LC–MS Analysis

LC–MS analysis was performed in an LC–MS 2020 system equipped with an LC10ADVP binary pump (Shimadzu, Japan). The sample was separated in a Phenomenex column (250 × 4.6 mm^2^, 5 μm) using acetonitrile (B)/water [(A) (0.1% formic acid)] as the mobile gradient phase. The injection volume was 20 μL, and the flow rate was set at 0.8 mL/min. Detection was done at a wavelength (λ) of 280 nm, with a run time of 20 min. The mass (MS) compartment consisted of a single quadrupole mass spectrometer with an electrospray ionization (ESI) source, and nitrogen gas was used to assist with nebulization at a flow rate of 1.5 L/min. The temperature was set for a curved desolation line (CDL) and heat blocks at 250 and 280 °C. All of the data were collected and processed using Lab Solution software (Shimadzu).

### 4.5 Analytical Characterization

This study examined the as-prepared materials using a PAN analytical X’pert pro-X-ray diffractometer equipped with a Cu K radiation (=1.54 Å) source at 40 kV and 40 mA at a temperature range of 2 = 5–70° using X-ray diffraction (XRD) patterns. With the support of Agilent Technologies, we were able to acquire the Fourier transform infrared (FT-IR) spectra of the solid sample embedded in the KBr pellets while the sample was still at room temperature. HR-TEM (200 keV, JEOL, JEM-2100f) and HR-SEM (America, FEI: NOVA Nano SEM 450) were employed to examine the morphology and microstructure of the structural applications. X-ray spectroscopy (XPS, EDXS, and ISIS300 Oxford) maps the ZrO_2_@MoS_2_/g-C_3_N_4_ nanocomposite surface profiles. The XPS binding energy data were calibrated using the carbon peak as a reference. The surface area and textural features were determined using an Autosorb IQ (quantachrome instruments version 5.0) and were assessed using the Brunauer–Emmett–Teller (BET) isotherm. The Barrett–Joyner–Halenda (BJH) method was used to determine pore size distributions. The catalyst’s diffuse reflectance spectroscopy (DRS) was carried out at room temperature using a Shimadzu UV 3600 plus in the 200–800 nm wavelength range to determine its UV–vis reflectance. PL measurements were taken with a Fluorolog (Horiba Yvon) spectrophotometer and recorded on a microfilm strip. A three-electrode setup was used to investigate the EIS response of all produced samples (Shanghai Chenhua CHI-660D). The photocatalytic performance evaluation was carried out with the help of a lamp source (Xe lamp, 300 (420 nm)). We used LC–MS analysis in the LC–MS 2020 system equipped with an LC10ADVP binary pump (Shimadzu, Japan).
